# Early Career Training Career Experiences and Insights in Dementia Care Science

**DOI:** 10.1093/geroni/igaf122.1505

**Published:** 2025-12-31

**Authors:** Joseph Gaugler, Allycia Wolff, Abraham Brody

## Abstract

Recent summits and national reports have emphasized the need for more rigorous methodological approaches when designing, evaluating, and disseminating/implementing dementia care interventions. The Advanced Behavioral Intervention Design in Dementia Care (ABIDDC) training and professional development program is designed to: 1) Deliver a graduate curriculum in advanced dementia care intervention science; 2) Establish a robust mentoring program to support early career investigators (pre-doctoral and post-doctoral fellows) in dementia care science; and 3) Leverage existing support at the University of Minnesota to position trainees to launch successful, independent careers in dementia care science. In addition to summarizing the ABIDDC program and its progress as a scientific training and professional development program, this symposium will summarize the training and mentoring experiences of current ABIDDC Fellows in similar initiatives to provide insights and recommendations to inform the refinement and creation of future scientific mentoring efforts in dementia care. Ultimately, this symposium will inform the infrastructure, training, and support necessary to cultivate a cadre of early career investigators and other investigators to conduct state-of-the-art dementia care science.

